# Repeated stimulation, inter-stimulus interval and inter-electrode distance alters muscle contractile properties as measured by Tensiomyography

**DOI:** 10.1371/journal.pone.0191965

**Published:** 2018-02-16

**Authors:** Hannah V. Wilson, Mark I. Johnson, Peter Francis

**Affiliations:** 1 Musculoskeletal Health Research Group, School of Clinical and Applied Science, Leeds Beckett University, Leeds, England, United Kingdom; 2 Centre for Pain Research, School of Clinical and Applied Science, Leeds Beckett University, Leeds, England, United Kingdom; The Ohio State University, UNITED STATES

## Abstract

**Context:**

The influence of methodological parameters on the measurement of muscle contractile properties using Tensiomyography (TMG) has not been published.

**Objective:**

To investigate the; (1) reliability of stimulus amplitude needed to elicit maximum muscle displacement (Dm), (2) effect of changing inter-stimulus interval on Dm (using a fixed stimulus amplitude) and contraction time (T_c_), (3) the effect of changing inter-electrode distance on Dm and T_c_.

**Design:**

Within subject, repeated measures.

**Participants:**

10 participants for each objective.

**Main outcome measures:**

Dm and T_c_ of the rectus femoris, measured using TMG.

**Results:**

The coefficient of variance (CV) and the intra-class correlation (ICC) of stimulus amplitude needed to elicit maximum Dm was 5.7% and 0.92 respectively. Dm was higher when using an inter-electrode distance of 7cm compared to 5cm [*P* = 0.03] and when using an inter-stimulus interval of 10s compared to 30s [*P* = 0.017]. Further analysis of inter-stimulus interval data, found that during 10 repeated stimuli T_c_ became faster after the 5^th^ measure when compared to the second measure [P<0.05]. The 30s inter-stimulus interval produced the most stable T_c_ over 10 measures compared to 10s and 5s respectively.

**Conclusion:**

Our data suggest that the stimulus amplitude producing maximum Dm of the rectus femoris is reliable. Inter-electrode distance and inter-stimulus interval can significantly influence Dm and/ or T_c_. Our results support the use of a 30s inter-stimulus interval over 10s or 5s. Future studies should determine the influence of methodological parameters on muscle contractile properties in a range of muscles.

## Introduction

Tensiomyography (TMG) is a non-invasive method used to measure spatial and temporal properties of muscle contraction. In response to an electrical stimulus, radial muscle belly displacement (millimetres) is measured by a digital displacement transducer, positioned perpendicular to the muscle belly. For each twitch response, 5 parameters are calculated from the displacement-time curve; contraction time (T_c_), delay time (T_d_), sustain time (T_s_) and relaxation time (T_r_) [1]. Dahmane et al. performed the first validation study and reported a strong association between T_c_ and percentage of type 1 muscle fibres (*r* = 0.93) [2]. Recent studies have demonstrated high within-day and inter-rater reliability for the measurement of muscle displacement (Dm) using TMG [3, 4]. A good to excellent agreement of Dm and T_c_ has been demonstrated for between week (ICC 0.62 and 0.86 respectively) and between-day reliability (ICC ≥ 0.98) [5, 6]. Perhaps due to their established validity and reliability and or their functional relevance, Dm and T_c_ are the most commonly investigated contractile properties.

**Fig 1:**
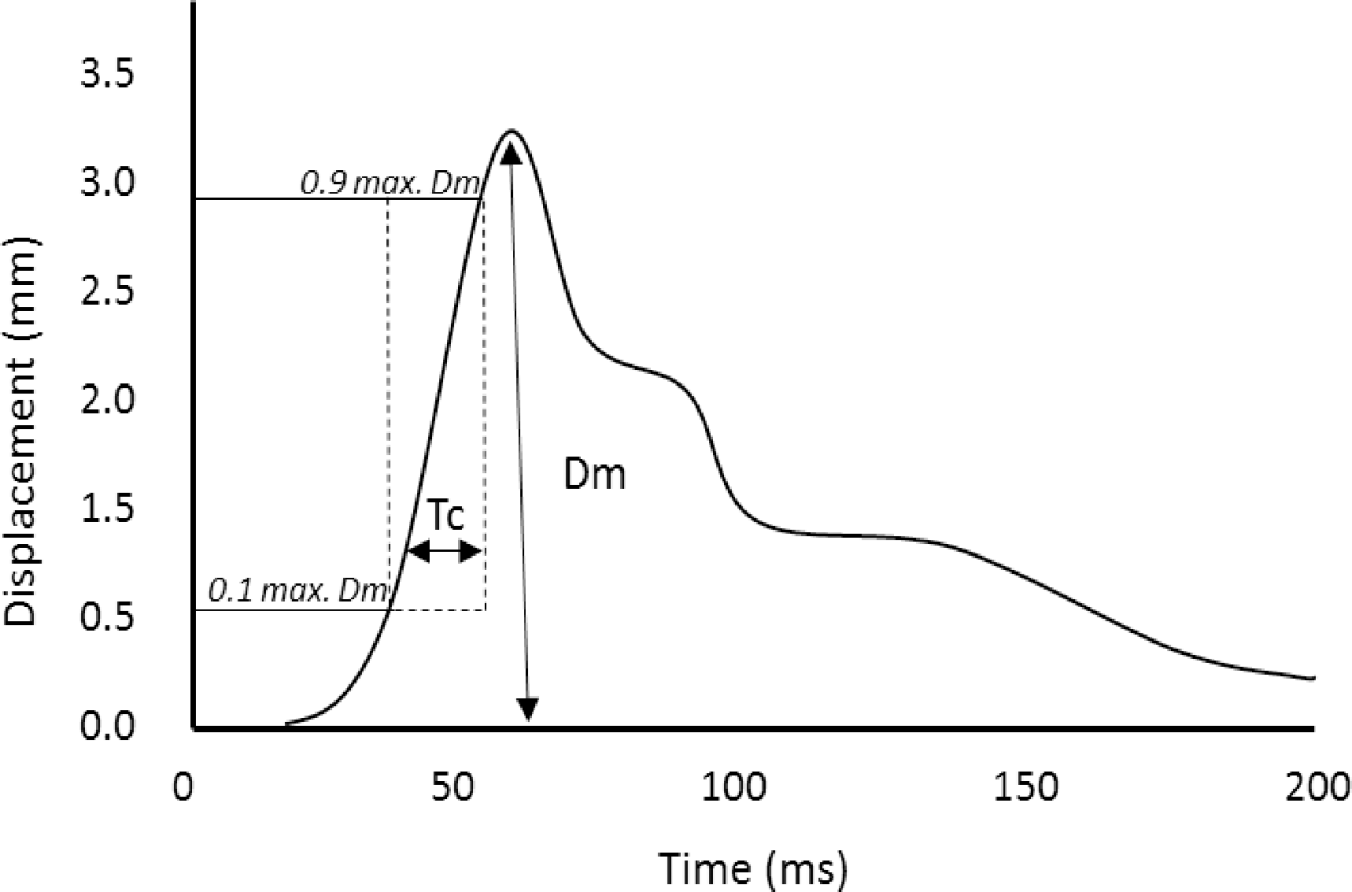
Typical Tensiomyography muscle contraction displacement-time curve. Contraction time (T_c_) is calculated as the time (ms) between 10% and 90% of the maximal Dm value of the displacement-time curve.

The displacement-time curve “Fig 1” is the product of 5 main properties of the electrical stimuli elicited by the TMG stimulator; stimulus waveform, stimulus duration, stimulus amplitude, inter-stimulus interval and inter-electrode distance. The TMG stimulator delivers a square stimulus waveform of 1ms stimulus duration, which cannot be modified by the user. Stimulus amplitude, inter-stimulus interval and inter-electrode distance can be modified by the user. However, it is not yet known whether changing inter-stimulus interval or inter-electrode distance effect Dm or T_c_ when using a fixed stimulus amplitude. Furthermore, during an intervention study, measures may be repeated at multiple time points; before, during and after an intervention [6–10]. Despite, earlier studies that have investigated TMG reliability, it is not yet understood if the stimulus amplitude of an individuals’ maximum Dm is reliable, or if a lower/ higher stimulus amplitude is required to elicit maximum Dm. Current practice among researchers to elicit maximum Dm, is to increase the stimulus amplitude from 20 or 30mA, in 10mA increments, until maximum Dm is achieved [6, 11–13]. Maximum Dm is typically determined by visualisation of overlapping displacement-time curves. In healthy participants, the normal range of stimulus amplitude needed for maximum Dm is between 40-70mA [3, 6, 14]. Therefore, to measure maximum Dm once, multiple consecutive stimulations are required, a process which is often repeated many times over within one experimental study. Short inter-stimulus intervals between consecutive stimulation may induce muscle fatigue. During electrical stimulation, fatigue may occur due to impaired calcium (Ca^2+^) release from the sarcoplasmic reticulum, increased myoplasmic Ca^2+^ and a reduced rate of cross-bridge kinetics [15]. More recently though, it has been found that although Ca^2+^ removal from the myoplasm is markedly reduced during fatigue, the effect of this is neutralised by reduced sensitivity of myofibrils to Ca^2+^. This suggests a reduced rate of cross-bridge detachment would be the most likely cause of muscle fatigue due to electrical stimulation [16]. Post activation potentiation may also develop due to short inter-stimulus intervals. Specifically, potentiation can develop due to phosphorylation of the regulatory myosin light chain, causing a transient increase in myosin-actin interaction [17]. Transient physiological conditions such as muscle fatigue and post activation potentiation could independently influence Dm and T_c_ measures. Written guidelines for a TMG operating procedure regarding stimulus schedule, specifically inter-stimulus interval, has not been published [18]. This has led to inconsistent inter-stimulus intervals used within previous literature, which typically range between 10-30s, or often not reported. TMG^TM^ collated a reference list of experimental studies and articles that have used TMG, between 1996 and 2013, from this we found 10s is the most frequently used inter-stimulus interval [3, 4, 19, 20]. It is not yet known whether repeated stimuli using TMG, with varied inter-stimulus intervals, alters Dm or T_c_ over time.

Electrode placement, specifically the inter-electrode distance, affects spatial recruitment of muscle fibres [21], which in turn may influence Dm and T_c_. Tous-Fajardo et al. reported a statistically significant decrease in Dm when reducing inter-electrode distance from 5cm to 3cm [4]. Although a standardised TMG protocol has not been published, investigator instructions within the TMG patent note that electrodes should be positioned 2-5cm from the point of measurement (probe position) [18]. From the TMG^TM^ reference list of experimental studies and articles, we found inconsistent inter-electrode distances used within previous literature, which typically ranged between 2-10cm [2, 12, 19, 22]. Many studies however report approximate inter-electrode distances or omit this information [6, 23–25]. The effect of inter-electrode distance on spatial and temporal TMG measures, Dm and T_c_, is uncertain and therefore there is a need to investigate the effect of inter-electrode distance on TMG measurements.

To the best of the authors’ knowledge there is no standard operating procedure for the use of TMG and the effect of varying inter-electrode distance and inter-stimulus interval on Dm and T_c_ is unknown. Additionally, reliability of stimulus amplitude needed for maximum Dm has not been previously assessed. The aims of this study were three fold:

1. to investigate the reliability of stimulus amplitude needed for maximum Dm,
2. to investigate the effect of changing inter-stimulus interval on Dm (using a fixed stimulus amplitude) and T_c_,
3. to investigate the effect of changing inter-electrode distance on Dm and T_c_.

## Materials and methods

### Design

A within subject, repeated measures study was designed using 3 distinct experiments:

1. The reliability of stimulus amplitude needed for maximum Dm.
2. The effect of changing inter-stimulus interval (30s, 10s or 5s) on Dm and T_c_.
3. The effect of changing inter-electrode distance (5cm, 7cm, 9cm or 11cm) on Dm and T_c_.

Participants could volunteer to participate in 1, 2 or 3 experiments. Ethical approval was obtained from 21 healthy volunteers (age: 27.0 ± 5.6 yr; height: 173.2 ± 8.3 cm; weight: 72.4 ± 11.9 kg). From the pool of 21 participants; 1 participant volunteered to for 3 experiments, 7 volunteered for 2 experiments and 13 volunteered for 1 experiment. Ethical approval was received from the Research Ethics Committee of Leeds Beckett University, and the study was conducted in accordance with the Helsinki Declaration for human research.

### Participant recruitment, screening and enrolment

Participants provided written informed consent before participating in the study. Participants were excluded if: <18 years; pregnant; taking medication; wearing an implantable medical device (e.g. pacemaker); did not consider themselves healthy; major long-term illness; lower limb and/or lower back injury (e.g. sciatica, muscle tear); experience disturbances to skin sensation (i.e. numbness, sensitivity or tingling) or have a dermatological condition(s) (e.g. dermatitis, eczema or bacterial/ fungal infection). Volunteers were given 48 hours before being formally invited to the laboratory. Volunteers were asked to refrain from participating in vigorous activity 72 hours before the laboratory visit to reduce the risk of developing delayed onset muscular soreness. Volunteers were also asked not to consume stimulants (e.g. caffeinated products) within 12 hours of the study visit.

Constrained block randomisation was used to allocate participants to an experiment. Participants selected one of three opaque envelopes. At the end of each experiment participants were reminded that they had participated in 1 of 3 experiments and asked if they would like to volunteer for another experiment within this study. If they agreed, the subsequent study visit was arranged to take place no less than 5 days after the previous study visit. Participants could not participate in the same experiment twice. Participants were reminded of their right to withdraw from the study at any point, without reason or consequence. Each experiment lasted no longer than 80 minutes and experimental instructions were standardized and read from a crib sheet.

### Experimental set-up

#### TMG set-up and general procedure

The rectus femoris muscle was chosen due frequent use within previous TMG experimental studies [14, 26–29] and the large size of the muscle which facilitated accurate palpation for TMG probe placement. The rectus femoris was stimulated using a TMG-S1 stimulator (EMF-Furlan and Co. d.o.o., Ljubljana, Slovenia) and radial muscle belly displacement was measured by a displacement transducer contained within a spring loaded probe (GK40, Panoptik d.o.o., Ljubljana, Slovenia). T_c_ was estimated from the displacement-time curve as the time between 10–90% of maximum Dm.

**Fig 2:**
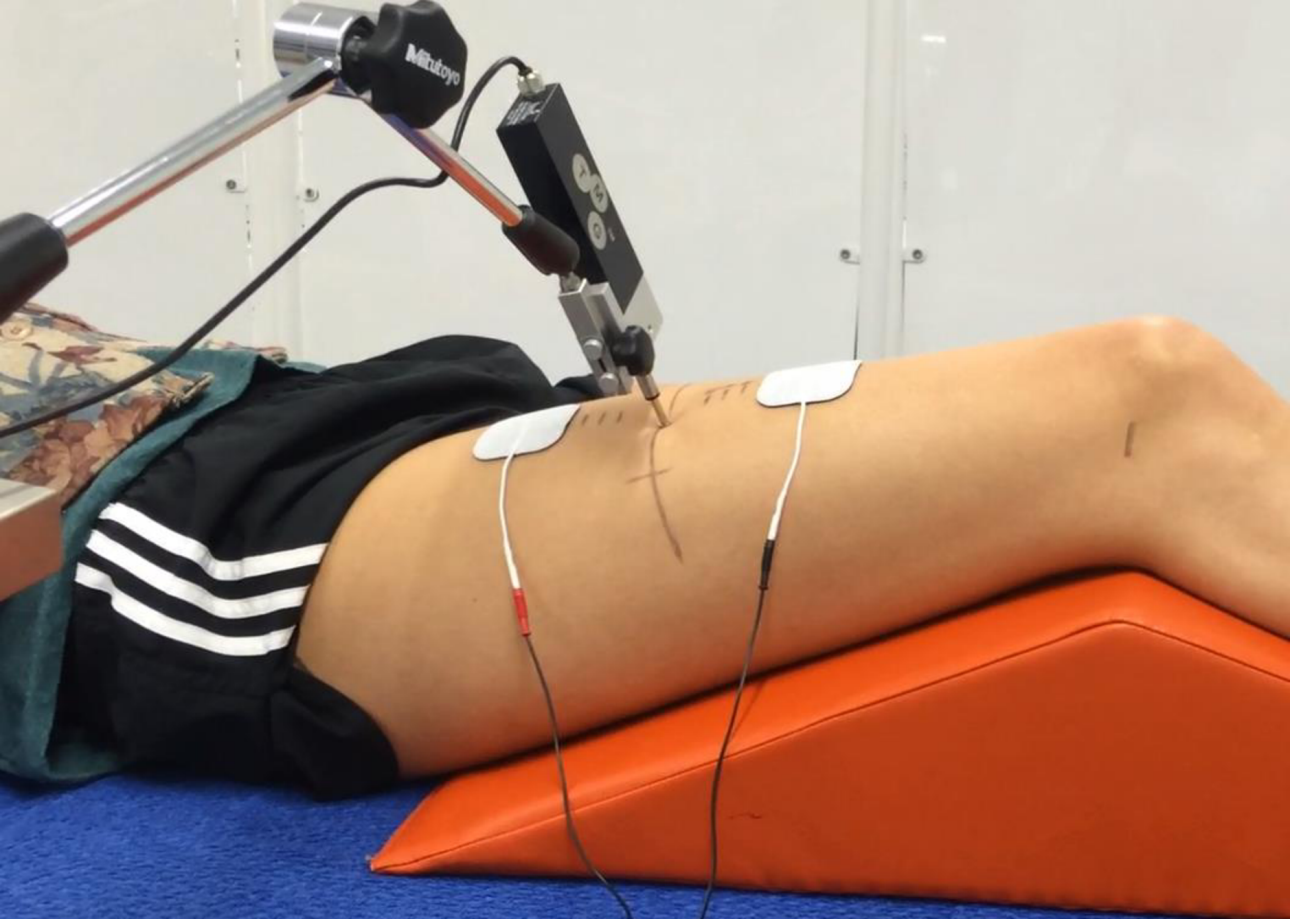
Tensiomyography set-up for assessment of muscle contractile properties. Knee flexed at 120°, relative to 180° full flexion.

Participants were positioned in the supine position and asked to relax with their arms to their sides, creating static and relaxed muscle conditions for TMG measures. A triangular foam pad was placed under the knee of the dominant leg (leg used to kick a ball) which supported knee-flexion at approximately 120° “Fig 2”. The probe was positioned perpendicular to the rectus femoris muscle belly which was determined using the following protocol; (i) using a dermatological pen, the skin marked at the midpoint between the greater trochanter and lateral femoral condyle, (ii) participants extend their knee against resistance from the investigator, while the investigator also palpated the rectus femoris muscle boarders and marked the skin at the midpoint between the medial and lateral borders, (iii) skin was marked “X” at the intersections of markings made during steps (i) and (ii) “Fig 2”.

The spring loaded probe of the displacement transducer was compressed into the muscle belly by 50%, this is estimated to create an initial pressure of approximately 1.5 X 10^-2^N/mm^2^ which has been reported to be controlled during experiments by retracting and repositioning the probe between measures [9]. Two square self-adhesive stimulating electrodes (5 x 5cm, Med-Fit, Stockport, UK) were positioned initially along the vertical axis with a 5cm gap between the leading edge of each electrode, one 2.5cm dorsal and one 2.5cm proximal to the probe.

A single, square electrical pulse stimulation, 1-millisecond-wide was delivered to the muscle. The stimulus amplitude, inter-electrode distance and inter-stimulus interval were dependent on the specific experiment (1, 2 or 3).

#### Experimental procedure

Participants were familiarized to TMG at the beginning of all experiments by verbal explanation and 2 muscle stimulations, starting at 20mA and then 30mA. A 2 minute wash out period was allowed before commencing experiments.

**Fig 3:**
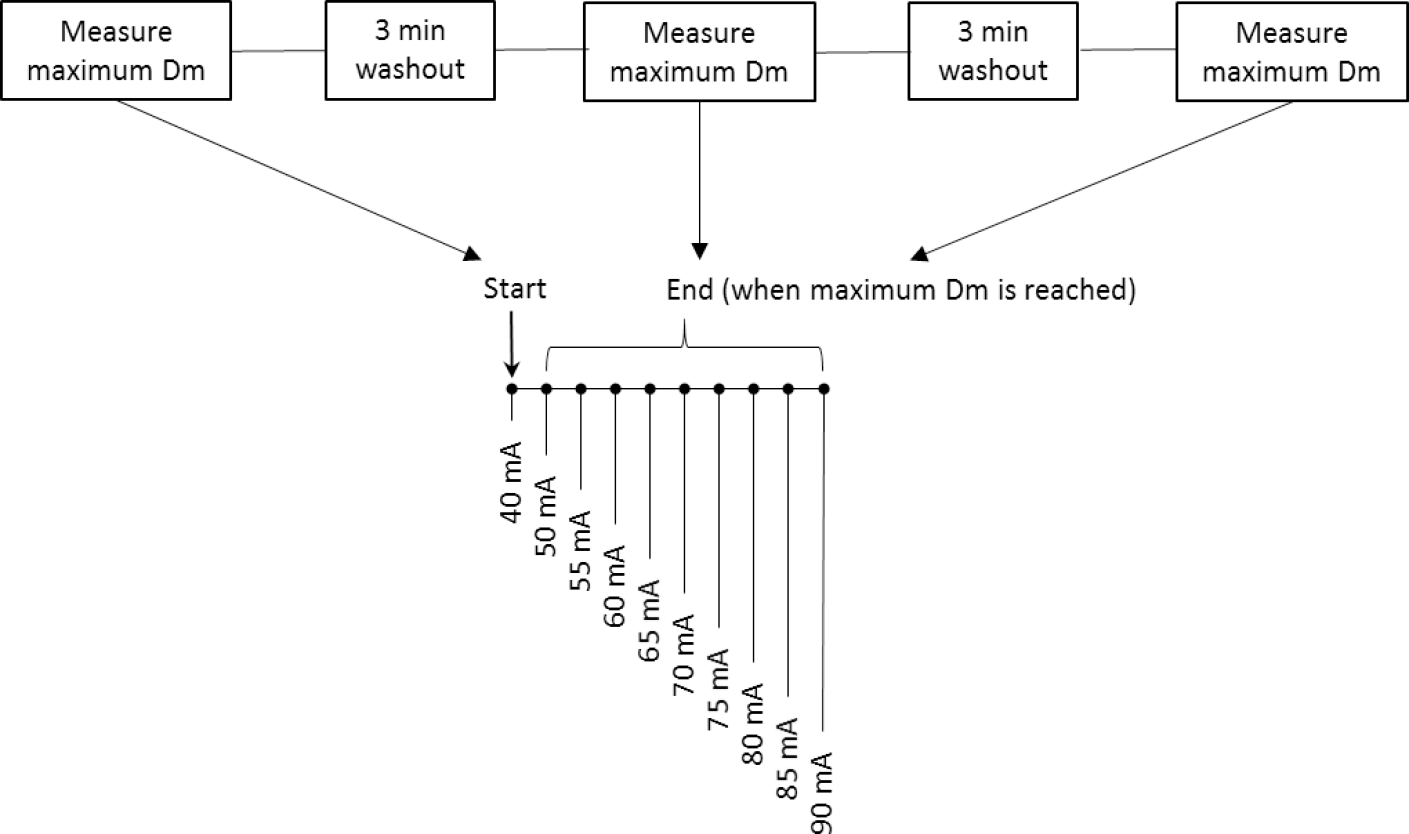
Experiment 1 timeline. The reliability of stimulus amplitude needed for maximum Dm. Tensiomyography measures are ceased when Dm is achieved. Stimulations are delivered at 1-minute intervals.

Fig 3 shows how experiment 1 was performed. The aim of experiment 1 was to assess the absolute and relative reliability between stimulus amplitude required to produce maximum Dm. Commencing at 40mA, amplitude was increased to 50mA and thereafter increased in increments of 5mA, every 1 minute until maximum Dm was reached. Ten milliamp increments were applied during amplitude ranges of submaximal Dm, and reduced to 5mA increments in amplitude ranges of maximum Dm, to keep overall stimulation counts to a minimum while maintaining sensitivity to determine the amplitude needed for maximum Dm. Maximum Dm was defined as the point of displacement plateau e.g. 50mA, 55mA and 60mA produce 3 overlapping displacement-time curves, the first amplitude in the sequence would be termed the amplitude of maximum Dm (e.g. 50mA). In cases where 3 overlapping displacement-time curves were not achieved, the highest displacement value before decline was used. This procedure was repeated 3 times for each participant.

**Fig 4:**
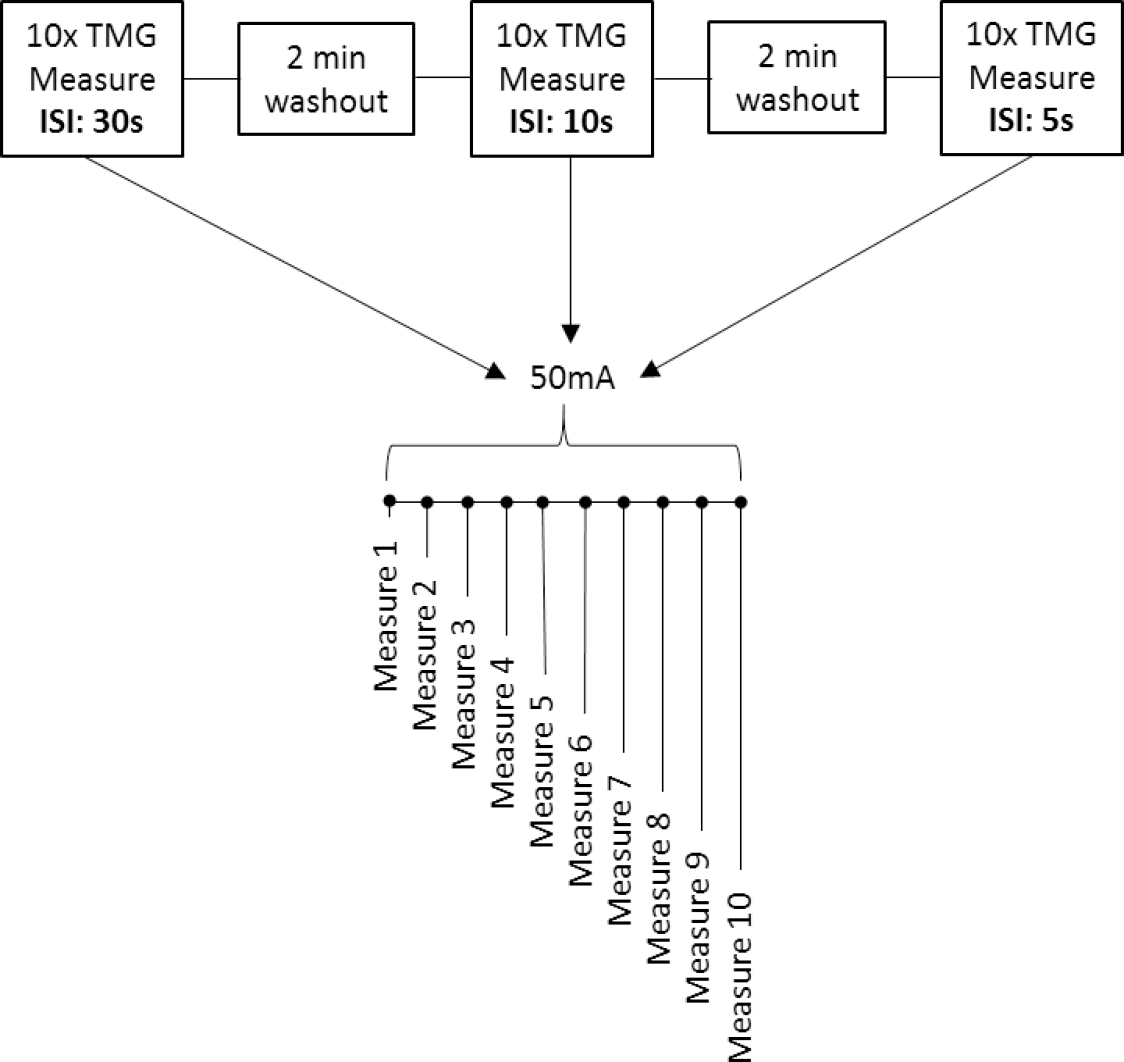
Experiment 2 timeline. Effect of changing inter-stimulus interval on Dm and T_c_. Abbreviation: ISI, inter-stimulus interval.

Fig 4 shows how this experiment 2 was performed. The aim of experiment 2 was to investigate if altering the time between consecutive stimuli, effects Dm and T_c_. For each inter-stimulus interval (30s, 10s and 5s), 10 consecutive measures were performed using a low intensity stimulus amplitude of 50mA, as previously used by Rodríguez-Matoso et al. [22]. Wash out periods of 2 minutes were given between inter-stimulus interval blocks of 10 measures and electrodes remained in situ throughout the experiment. Measurements were recorded sequentially for inter-stimulus intervals commencing with 30s, then 10s and finally 5s. Although 10 consecutive stimulations using the same stimulus amplitude would be seldom used in experimental studies, this study design was necessary to assess the effect of inter-stimulus interval over 10 repeated measures on Dm and T_c_.

**Fig 5:**
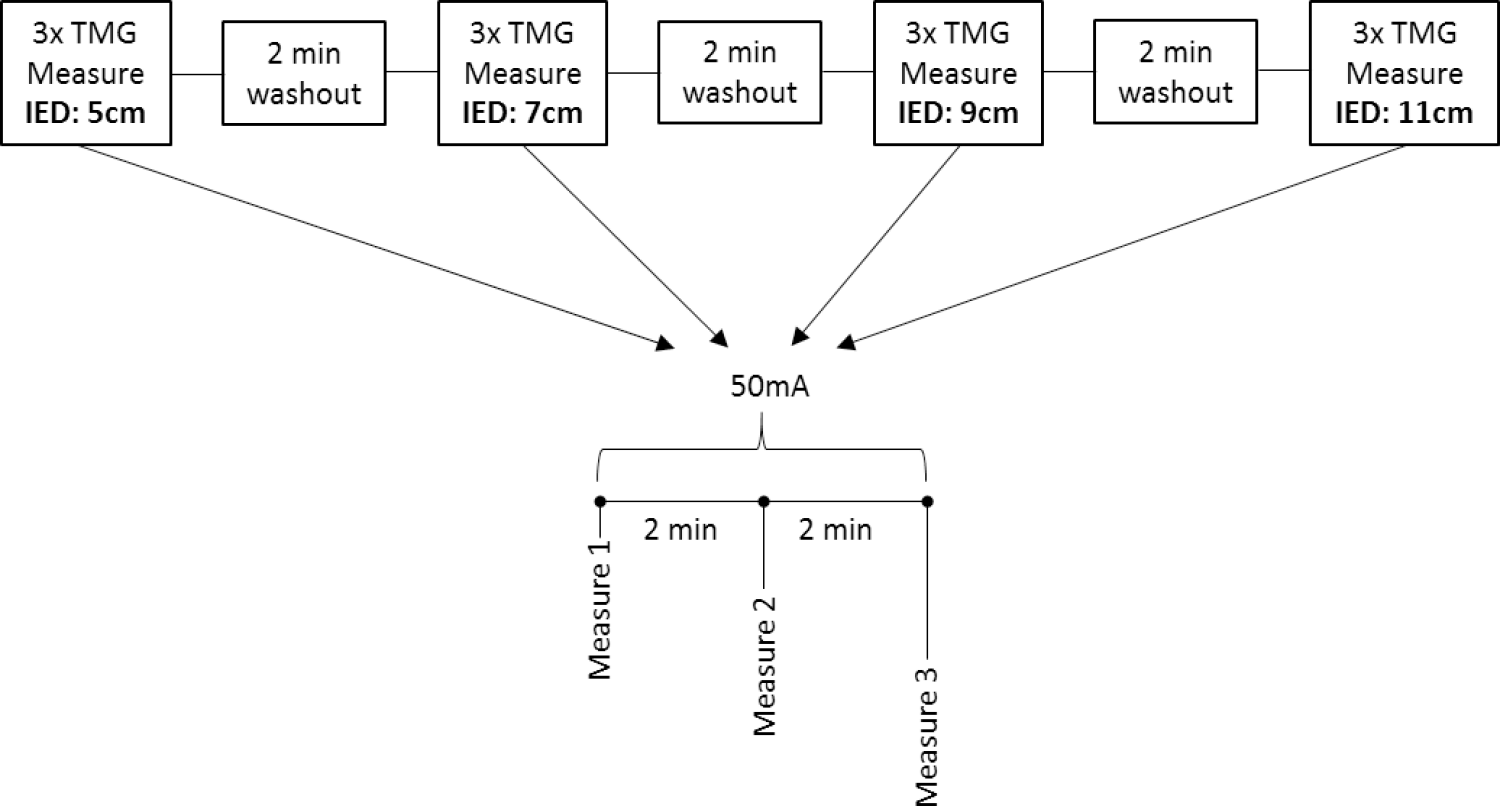
Experiment 3 timeline. The effect of changing inter-electrode distance on Dm and T_c_. Abbreviation: IED, inter-electrode distance.

Fig 5 shows how this experiment 3 was performed. The aim of experiment 3 was to investigate if altering the distance between electrodes, effects Dm and T_c_ values. Three consecutive TMG measures were performed for each inter-electrode distance, using a low intensity stimulus amplitude of 50mA. Stimulations were delivered every 2 minutes and electrodes remained in situ between consecutive measures of the same inter-electrode distance. Measurements were recorded sequentially for inter-electrode distances, commencing with 5cm, then 7cm, then 9cm, and finally 11cm.

### Data management and analysis

Data was assessed for normality using a Shapiro-Wilk test. Descriptive statistics are reported as mean (M) ± standard deviation (SD). To assess the absolute and relative reliability of stimulus amplitude required to achieve three repeated measures of maximum Dm, the percentage coefficient of variation (CV) and intra-class correlation (ICC) were calculated. Relative CV was calculated as (SD of repeated measures/ mean of repeated measures) * 100. For this study, an ICC <0.50, between 0.50–0.75, between 0.75–0.90 and >0.90 were considered poor, moderate, good and excellent respectively. Regarding CV, <10% was considered a good relative reliability. To assess the effect of changing inter-stimulus interval (30s, 10s, 5s) on Dm and T_c_, a within-subject repeated measures multifactorial, 3 (inter-stimulus interval: 30s, 10s and 5s) x 10 (repeated stimuli: measure 1 to measure 10) ANOVA was performed. To assess the effect of changing inter-electrode distance (5cm, 7cm, 9cm and 11cm) on Dm and T_c_, a simple within-subject repeated measures analysis of variance (ANOVA) was performed. Statistical significance was defined as *P* = <0.05. Where significant differences were found, a pairwise comparison with Bonferroni correction for multiple comparisons, was used to identify where differences occurred. Statistical analyses were conducted using IBM SPSS statistical package for Windows (version 23).

## Results

The mean CV and ICC of stimulus amplitude needed to elicit maximum Dm were 5.7% and 0.92 respectively.

**Fig 6:**
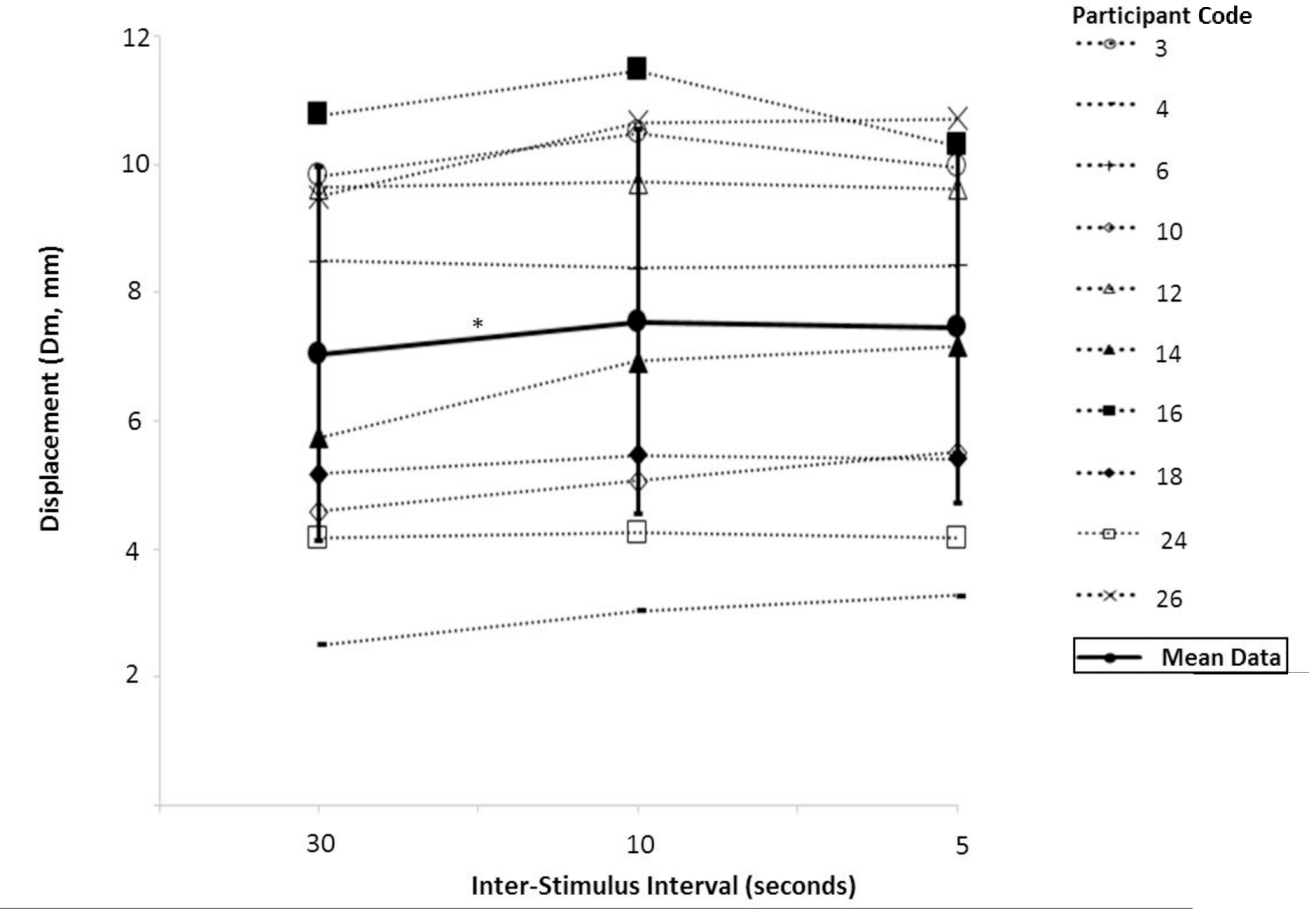
Dm measured using Tensiomyography at four inter-electrode distances. Mean and individual data for each of the 10 participants is presented. *Significant difference between groups, *P* <0.05.

Inter-stimulus interval had a statistically significant effect on Dm [P = 0.014, F = 5.414, df = 2] “Fig 6”. Dm was higher when using an inter-stimulus interval of 10s (M; 7.545, SD; 2.870) compared to 30s (M; 7.041, SD; 2.788) [P = 0.017]. Dm was not different between 30s and 5s (M; 7.454, SD; 2.603) [P = 0.200] or 10s and 5s [P = 1.000]. Inter-stimulus interval did not have a statistically significant effect on T_c_ [P = 0.062, F = 4.450, df = 1.047].

Repeated stimuli, of which there were 10, had no statistically significant effect on Dm [P = 0.090, F = 2.65, df = 2.316]. Compared to measure 2 (M; 26.982, SD; 4.358), measures 5 (M; 26.441, SD; 4.086), 6 (M; 26.343, SD; 4.231), 7 (M; 26.140, SD; 4.046) and 9 (M; 25.898, SD; 3.856) were significantly faster [P >0.05] during 10 repeated stimuli. There was no interaction between inter-stimulus interval and repeated muscle stimulation for Dm [P = 0.735] or T_c_ [P = 0.258]. To further assess the relationship between 10 repeated stimuli and T_c_, CV was calculated for the mean of 10 repeated measures, for each inter-stimulus interval. The CV of T_c_ for 30s, 10s and 5s inter-stimulus intervals was 1%, 2% and 2% respectively.

**Fig 7:**
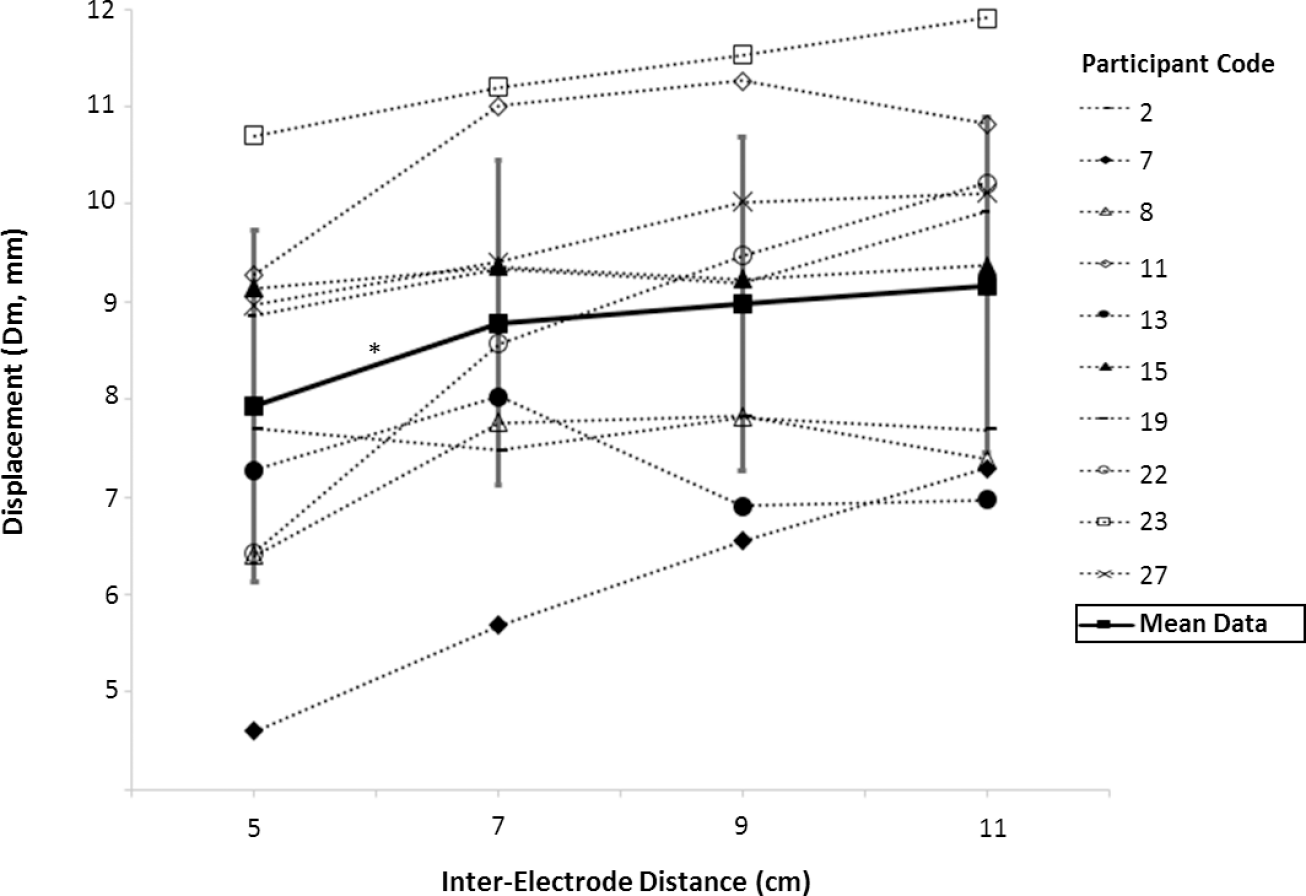
Dm measured using Tensiomyography at three inter-stimulus intervals. Mean and individual data for each of the 10 participants is presented. *Significant difference between groups, *P* <0.05.

Inter-electrode distance had a statistically significant effect on Dm [*P* = 0.008, F = 7.956, df = 1.494] “Fig 7”. Dm was higher when using an inter-electrode distance of 7cm (M; 8.782, SD; 1.659) compared to 5cm (M; 7.934, SD; 1.798) [*P* = 0.03], but did not alter between 7–11cm [P <0.05]. Inter-electrode distance had no effect on T_c_ [*P* = 0.132, F = 2.036, df = 3].

## Discussion

Methodological variability exists within previous literature when measuring maximum Dm using TMG, specifically in relation to stimulus amplitude, inter-stimulus interval and inter-electrode distance [11, 30–32]. Absolute and relative reliability for the stimulus amplitude required to achieve three repeated measures of maximum Dm were considered good (CV; <10%) and excellent (ICC; >0.90) respectively [33]. Dm increased when inter-stimulus interval was reduced from 30s to 10s. Overall T_c_ appeared to decrease i.e. the contraction became faster, after the 5^th^ of 10 repeated stimulations when compared to measure 2, however this was with the exception of measures 8 and 10. Further analysis of the relationship between 10 repeated stimuli and T_c_ revealed the 30s inter-stimulus interval produced the lowest CV and therefore, although not statistically significant, 30s produced the numerically the most stable T_c_ compared to 10s and 5s. The least stable T_c_ occurred using an inter-stimulus interval of 5s. Finally, Dm increased when inter-electrode distance was increased from 5cm to 7cm but did not change further when increased to 11cm.

Unlike previous studies which have assessed the reliability of individual contractile properties, i.e. Dm, T_c_, T_d_, T_s_ and T_r_, our study assessed the reliability of the stimulus amplitude needed to elicit maximum Dm. This was important because a different stimulus amplitude may result in an equivalent Dm i.e. a left or right shift in the dose-response curve where, dose is represented by stimulus amplitude and response is represented by Dm. Within intervention studies, specifically interventions designed to facilitate muscle contraction, a leftward shift in the dose-response curve may be misinterpreted as a false positive. Although our findings suggest that there is a good absolute and excellent relative reliability, the amplitude needed for maximum Dm can vary as much as ~6% on average in a small sample of 10 participants. Researchers should be aware of this when assessing change in muscle contractile properties using TMG. It may be important to record change in stimulus amplitude alongside change in Dm, to fully understand changes observed.

The finding of higher Dm values using an inter-stimulus interval of 10s compared to 30s demonstrates the need for a definitive TMG stimuli scheme regarding inter-stimulus interval. The lack of defined inter-stimulus interval has led to substantial variation of inter-stimulus intervals used in current literature [4, 11, 30, 32, 34]. Our study suggests an increase in Dm is associated with a shorter inter-stimulus interval, which may be explained by the phenomenon of post activation potentiation. Post activation potentiation is characterised by a transient increase in mechanical performance due to elevated cross-bridge cycling. This effect is associated with increased sensitivity of the contractile proteins to extracellular Ca^2+^, due to phosphorylation of regulatory myosin light chains [35]. Furthermore, during post activation potentiation, the increase in twitch amplitude i.e. Dm amplitude, is accompanied by either a decreased T_c_ i.e. the contraction becomes faster [36], or is unchanged [37]. This phenomenon could explain results of this study, as Dm increased with shorter inter-stimulus intervals and no significant effect on T_c_ was found. The extent to which post activation potentiation may develop is influenced by muscle fibre composition and the intensity and duration of the conditioning contraction [38]. Within this study however, the effects of inter-stimulus interval were assessed within one muscle only (rectus femoris) and both the stimuli amplitude and stimuli width were constant, 50mA and 1ms respectively. Therefore, it is reasonable to suggest that the effects observed on Dm amplitude were due to the variation in inter-stimulus interval.

Post activation potentiation may also explain why T_c_ over 10 repeated stimuli, on the whole became significantly faster after the 5^th^ stimuli which was irrespective of inter-stimulus interval. We therefore suggest that neither 5s, nor 10s, nor 30s are long enough inter-stimulus intervals to completely eliminate post activation potentiation during repeated stimuli protocols using TMG. However, the 30s inter-stimulus interval demonstrated the smallest T_c_ variance from 10 repeated stimuli, in addition to eliciting a statistically smaller Dm compared to 10s.

Finally, we consider the potential influence of muscle fatigue on our data which is characterised by decreased Ca^2+^ concentration in the myoplasm, a decreased myofilament sensitivity to Ca^2+^ and a decline in contractile response i.e. Dm would decline and T_c_ would increase if fatigue was in effect [39]. This effect has been demonstrated by Westerblad and Allen [16] who reported slowed post tetani relaxation in association with slowed cross-bridge kinetics. Our study however observed opposing effects of numerically higher Dm amplitudes and faster T_c_ with 5s compared to numerically lower Dm amplitudes and slower T_c_ with 30s inter-stimulus interval. Furthermore, recovery after fatigue produced by repeated tetani is a slow process which would not be indicative of our finding that repeated stimuli elicited faster contractions during repeated stimuli over time. It is therefore unlikely that our results reflect the effects of muscle fatigue and overall we suggest that our results reflect the effects of post activation potentiation.

The increase in Dm when inter-electrode distance is increased from 5cm to 7cm extends the findings of Tous-Fajardo et al. who reported an increase in Dm when electrode distance was increased from 3cm to 5cm [4]. This is an important finding as manufacturer guidelines recommend an electrode distance of 2-5cm and 5cm is most commonly used within previous literature. Electrode arrangement and inter-electrode distance significantly affected muscle contraction, contractile onset latency (time taken for the conduction of sensory fibres) and conduction amplitude [40, 41]. Previous literature suggests that increased Dm, due to increase inter-electrode distance, may be due to increased muscle fibre recruitment [13]. It is known that superficial and larger diameter neurons are the first to be recruited via electrical stimulation [42]. It is therefore logical to suggest that as distance between surface electrodes increases, the number of motor nerve fibres that are stimulated will increase. In turn, this will increase the number of depolarising muscle fibres and increase the degree of muscle contraction. Intrinsic factors such as excitation threshold, skin conductivity, water retention, temperature, subcutaneous fat thickness, muscle stiffness, fascia thickness, fibre composition (proportions of fast and slow twitch muscle fibres), elastic properties of the intramuscular connective tissue, motor neuron branching/ orientation and location will also influence mean Dm [3, 21, 24].

Our findings need to be interpreted cognisant of the following limitations. Participant numbers are low, N = 10 per experimental group. The orders of conditions (inter-stimulus interval and inter-electrode distance) were not randomised, thus there was a potential for progressive error and carry over effects. However, to limit this we allowed extensive washout periods between conditions.

Findings of this study support the reliability of TMG measures, when performed in conjunction with probe withdrawal and repositioning between TMG measures. Current TMG guidelines state that a 2-5cm inter-electrode distance should be used, this distance is often judged by eye and not measured precisely. We suggest for the rectus femoris, that if 5cm is used, accurate measurement is essential and an appreciation of the effect inter-electrode distance has on Dm between 5cm and 7cm. Care should be taken when comparing experimental findings, arising from varying inter-electrode distances. To the authors’ knowledge, no current guidelines exist for inter-stimulus interval, however current literature most commonly uses 10s. In line with the findings of this study, we recommend a 30s inter-stimulus interval should be employed, over the use of 10s or 5s. This study has contributed towards understanding the impact of the variability in TMG protocols used in previous literature, on TMG measures. This information may be used to develop a TMG standard operating procedure such that experimental studies may be comparable. It should be acknowledged however that TMG is used to measure a wide range of superficial muscles of varying size and shape. As well as the rectus femoris, frequently investigated muscles are the biceps femoris [19, 20, 27], biceps brachii [3, 43, 44] and gastrocnemius [6, 9, 34]. It is therefore important to investigate the impact of methodological variability using TMG, in larger sample sizes and a wider range of superficial muscles, to develop muscle specific TMG standard operating procedures.
